# *catena*-Poly[[aqua­(benzoato-κ^2^ *O*,*O*′)(benzoic acid-κ*O*)calcium]-μ_3_-benzoato-κ^4^ *O*:*O*,*O*′:*O*′]

**DOI:** 10.1107/S1600536811013493

**Published:** 2011-04-16

**Authors:** Olimjon Azizov, Zukhra Kadirova, Tohir Azizov, Samat Tolipov, Bakhtiyar Ibragimov

**Affiliations:** a Institute of General and Inorganic Chemistry, Abdullaev St 32, Tashkent 100077, Uzbekistan; b Tashkent Chemical–Technological Institute, Navoi St 32, Tashkent 100011, Uzbekistan; c Institute of Biorganic Chemistry, Mirzo-Ulugbek St 83, Tashkent 100125, Uzbekistan

## Abstract

In title compound, [Ca(C_7_H_5_O_2_)_2_(C_7_H_6_O_2_)(H_2_O)]_*n*_, the eightfold-coordinated Ca^II^ ion is bonded to four carboxyl­ate O atoms from two benzoate ions, an O atom from benzoic acid and a water O atom. One of the carboxyl­ate groups bridges adjacent Ca^2+^ ions, forming a polymeric ribbon structure parallel to [010]. In the crystal, the benzoate anions and water mol­ecule inter­act by way of inter- and intra­molecular O—H⋯O hydrogen bonds.

## Related literature

For background to the crystal structures and physical stability of calcium benzoate hydrates, mesophases and related compounds, see: Cherkezova *et al.* (1987 ▶); Zhang *et al.* (1999 ▶); Yano *et al.* (2001 ▶); Senkovska & Thewalt (2005 ▶); Terakita & Byrn (2006 ▶).
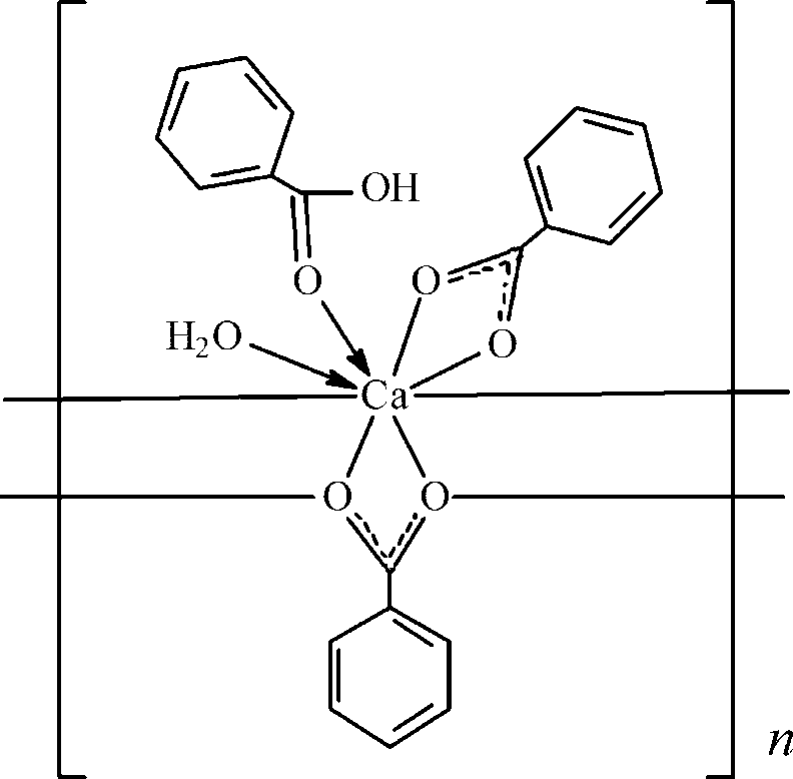


## Experimental

### 

#### Crystal data

[Ca(C_7_H_5_O_2_)_2_(C_7_H_6_O_2_)(H_2_O)]
*M* *_r_* = 422.43
Monoclinic, 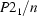

*a* = 15.5535 (3) Å
*b* = 6.61183 (16) Å
*c* = 20.1828 (4) Å
β = 94.3750 (18)°
*V* = 2069.49 (8) Å^3^
*Z* = 4
Cu *K*α radiation
μ = 2.96 mm^−1^
*T* = 293 K
0.55 × 0.45 × 0.40 mm

#### Data collection

Oxford Diffraction Xcalibur Ruby diffractometer
Absorption correction: multi-scan (*CrysAlis PRO*; Oxford Diffraction, 2007 ▶) *T*_min_ = 0.782, *T*_max_ = 1.000
7257 measured reflections
3847 independent reflections
2961 reflections with *I* > 2σ(*I*)
*R*_int_ = 0.025

#### Refinement

*R*[*F* ^2^ > 2σ(*F* ^2^)] = 0.041
*wR*(*F* ^2^) = 0.121
*S* = 1.07
3847 reflections
275 parameters
H atoms treated by a mixture of independent and constrained refinement
Δρ_max_ = 0.27 e Å^−3^
Δρ_min_ = −0.26 e Å^−3^

### 

Data collection: *CrysAlis PRO* (Oxford Diffraction, 2007 ▶); cell refinement: *CrysAlis PRO*; data reduction: *CrysAlis PRO*; program(s) used to solve structure: *SHELXS97* (Sheldrick, 2008 ▶); program(s) used to refine structure: *SHELXL97* (Sheldrick, 2008 ▶); molecular graphics: *ORTEP-3 for Windows* (Farrugia, 1997 ▶); software used to prepare material for publication: *publCIF* (Westrip, 2010 ▶).

## Supplementary Material

Crystal structure: contains datablocks I, global. DOI: 10.1107/S1600536811013493/bv2180sup1.cif

Structure factors: contains datablocks I. DOI: 10.1107/S1600536811013493/bv2180Isup2.hkl

Additional supplementary materials:  crystallographic information; 3D view; checkCIF report

## Figures and Tables

**Table 1:** Hydrogen-bond geometry (Å, °)

*D*—H⋯*A*	*D*—H	H⋯*A*	*D*⋯*A*	*D*—H⋯*A*
O1*W*—H1*W*1⋯O5^i^	0.76 (3)	2.05 (3)	2.779 (2)	163 (3)
O1—H1*O*⋯O6	0.93 (3)	1.68 (3)	2.597 (2)	167 (3)
O1*W*—H2*W*1⋯O6^ii^	0.89 (3)	1.90 (3)	2.754 (2)	159 (3)

Symmetry codes: (i) 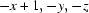; (ii) 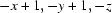.

**Fig. 1.**
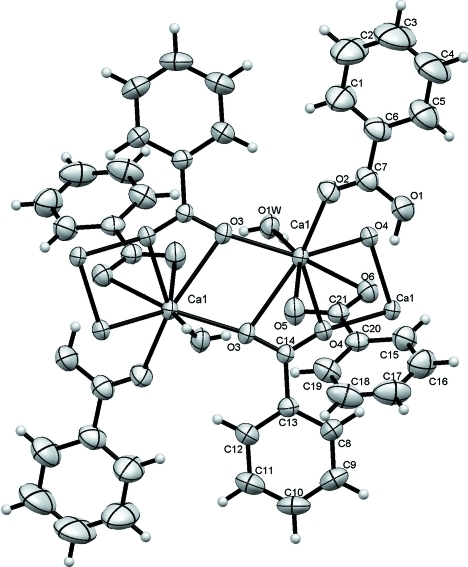
A view of the structure of (I), showing the atom numbering scheme. Displacement ellipsoids are drawn at the 50% probability level and H atoms are shown as small spheres of arbitary radii.

**Fig. 2.**
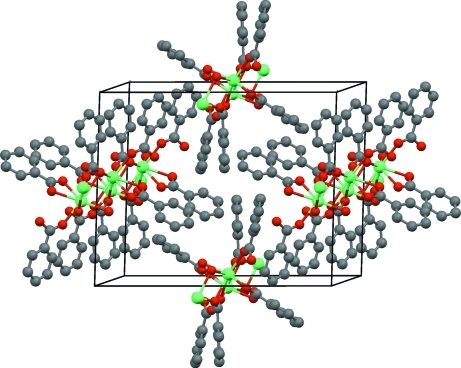
The crystal structure packing scheme showing the hydrogen bonds system.

## supplementary crystallographic information

### Comment

The synthesis and structure determination of inorganic polymers are interesting subject for basic inorganic chemistry and materials science. Depending on the pH and other synthetic conditions, many calcium benzoates with different coordination modes, polymeric arrangements and molecular topologies have been observed, *e.g.* [Ca(C_6_H_5_COO)_2_]×3H_2_O(neutral solution; Terakita *et al.*, 2006), Ca(C_6_H_5_COO)_2_](C_6_H_5_COO)_0.5_×2H_2_O (acid solution; Cherkezova *et al.*, 1987), [Ca(C_6_H_5_COO)(H_2_O)_3_](C_6_H_5_COO)]_n_ (basic solution; Senkovska *et al.*, 2005),[Ca(C_6_H_5_COO)_2_(C_3_H_7_NO)(H_2_O)]_n_ (dimetylformamide solution; Yano *et al.*, 2001), [Ca(C_6_H_5_COO)_2_] (hydrothermal conditions; Zhang *et al.*, 1999).

In this study we synthesized the Ca^II^ polymeric compound, (I), bridged by a benzoate group, and report the structure of the title compound, (I). The molecular structure is shown on Fig.1 and geometrical parameters are available from archived CIF.

The asymmetric unit of (I) consists of one Ca centre, two benzoate anions, benzoic acid and one water molecule (Fig 1). The calcium ion is surrounded by eight O atoms from two tri- and bidentate benzoates, a monodentate benzoic acid molecule, and a water molecule. The CaO_8_ polyhedron deviates extensively from idealized octacoordinated geometries found in other complexes (Senkovska *et al.*, 2005; Yano *et al.*, 2001). There are three different coordination modes of benzoic acid in crystal structure. The tridentate benzoate forms simultaneously the planar four-membered chelate and the buckled four-membered Ca–O–Ca–O rings by bridging adjacent Ca^2+^ ions. The Ca–O bridging bond lengths [2.3204 (14) and 2.3781 (14) Å] are considerably shorter than the Ca–O chelate distances [2.7414 (14) and 2.4567 (14) Å]. The bidentate benzoate has longer Ca–O distances [2.4837 (17) and 2.5628 (15) Å] than observed for monodentate benzoic acid and calcium ion [2.4467 (15) Å].

The bridging interactions and the system of H-bonds form polymeric structure consisted from the infinite ribbons along the  *b* axis and separated by the stacked neighbouring phenyl groups. The benzoic acid hydroxyl group and an water molecule act as H-bond donors, and the O5 and O6 atoms of the bidentate COO^-^ group are H-bond acceptors. The combination of these hydrogen bonds, *π-π* stacking interactions and the Ca–O bonds leads to the formation of a two-dimensional network running parallel to the *ac-*plane (Fig. 2).

### Experimental

The Ca(NO_3_)_2_×4H_2_O (1 mmol) and benzoic acid (3 mmol) in 75 ml of ethanol were mixed with the the benzoic acid water solution (2 mmol). The mixture were strirred 6 h at room temperature, and after 3 days the precipitated colourless crystals were filtered off, washed three times with ethanol, dried at room temperature. Crystals of the title compound, suitable to X-ray diffraction analysis, were selected directly from the sample as prepared.

### Refinement

All e.s.d.'s (except the e.s.d. in the dihedral angle between two l.s. planes) are estimated using the full covariance matrix. The cell e.s.d.'s are taken into account individually in the estimation of e.s.d.'s in distances, angles and torsion angles; correlations between e.s.d.'s in cell parameters are only used when they are defined by crystal symmetry. An approximate (isotropic) treatment of cell e.s.d.'s is used for estimating e.s.d.'s involving l.s. planes.

Refinement of F^2^ against ALL reflections. The weighted *R*-factor *wR* and goodness of fit S are based on F^2^, conventional *R*-factors *R* are based on F, with F set to zero for negative F^2^. The threshold expression of F^2^ > 2sigma(F^2^) is used only for calculating *R*-factors(gt) *etc*. and is not relevant to the choice of reflections for refinement. *R*-factors based on F^2^ are statistically about twice as large as those based on F, and R– factors based on ALL data will be even larger.

All the H-atoms were found in the difference Fourier synthesis and refined with restrained O–H 0.82 (2) Å, H···H 1.35 (2) Å, but free isotropic displacement parameters.

### Crystal data

**Table:**

[Ca(C_7_H_5_O_2_)_2_(C_7_H_6_O_2_)(H_2_O)]	*F*(000) = 880
*M**_r_* = 422.43	*D*_x_ = 1.356 Mg m^−^^3^
Monoclinic, *P*2_1_/*n*	Cu *K*α radiation, λ = 1.54184 Å
Hall symbol: -P 2yn	Cell parameters from 3133 reflections
*a* = 15.5535 (3) Å	θ = 3.5–70.6°
*b* = 6.61183 (16) Å	µ = 2.96 mm^−^^1^
*c* = 20.1828 (4) Å	*T* = 293 K
β = 94.3750 (18)°	Monoclinic, colourless
*V* = 2069.49 (8) Å^3^	0.55 × 0.45 × 0.40 mm
*Z* = 4	

### Data collection

**Table:**

Oxford Diffraction Xcalibur Ruby diffractometer	3847 independent reflections
Radiation source: Enhance (Cu) X-ray Source	2961 reflections with *I* > 2σ(*I*)
graphite	*R*_int_ = 0.025
Detector resolution: 10.2576 pixels mm^-1^	θ_max_ = 71.1°, θ_min_ = 3.5°
q/2θ scans	*h* = −17→18
Absorption correction: multi-scan (*CrysAlis PRO*; Oxford Diffraction, 2007)	*k* = −7→7
*T*_min_ = 0.782, *T*_max_ = 1.000	*l* = −22→24
7257 measured reflections	

### Refinement

**Table:**

Refinement on *F*^2^	Secondary atom site location: difference Fourier map
Least-squares matrix: full	Hydrogen site location: inferred from neighbouring sites
*R*[*F*^2^ > 2σ(*F*^2^)] = 0.041	H atoms treated by a mixture of independent and constrained refinement
*wR*(*F*^2^) = 0.121	*w* = 1/[σ^2^(*F*_o_^2^) + (0.0754*P*)^2^] where *P* = (*F*_o_^2^ + 2*F*_c_^2^)/3
*S* = 1.07	(Δ/σ)_max_ < 0.001
3847 reflections	Δρ_max_ = 0.27 e Å^−^^3^
275 parameters	Δρ_min_ = −0.26 e Å^−^^3^
0 restraints	Extinction correction: *SHELXL97* (Sheldrick, 2008), Fc^*^=kFc[1+0.001xFc^2^λ^3^/sin(2θ)]^-1/4^
Primary atom site location: structure-invariant direct methods	Extinction coefficient: 0.0050 (4)

### Special details

**Table:**

Geometry. All e.s.d.'s (except the e.s.d. in the dihedral angle between two l.s. planes) are estimated using the full covariance matrix. The cell e.s.d.'s are taken into account individually in the estimation of e.s.d.'s in distances, angles and torsion angles; correlations between e.s.d.'s in cell parameters are only used when they are defined by crystal symmetry. An approximate (isotropic) treatment of cell e.s.d.'s is used for estimating e.s.d.'s involving l.s. planes.Bond distances, angles *etc*. have been calculated using the rounded fractional coordinates. All su's are estimated from the variances of the (full) variance-covariance matrix. The cell e.s.d.'s are taken into account in the estimation of distances, angles and torsion angles
Refinement. Refinement of *F*^2^ against ALL reflections. The weighted *R*-factor *wR* and goodness of fit *S* are based on *F*^2^, conventional *R*-factors *R* are based on *F*, with *F* set to zero for negative *F*^2^. The threshold expression of *F*^2^ > σ(*F*^2^) is used only for calculating *R*-factors(gt) *etc*. and is not relevant to the choice of reflections for refinement. *R*-factors based on *F*^2^ are statistically about twice as large as those based on *F*, and *R*- factors based on ALL data will be even larger.

### Fractional atomic coordinates and isotropic or equivalent isotropic displacement parameters (Å^2^)

**Table:**

	*x*	*y*	*z*	*U*_iso_*/*U*_eq_	
Ca1	0.45197 (2)	0.25532 (5)	0.033347 (19)	0.03194 (16)	
O1W	0.39727 (11)	0.2332 (3)	−0.08057 (9)	0.0443 (4)	
O1	0.36276 (12)	0.5693 (3)	0.16770 (10)	0.0675 (6)	
O2	0.33262 (10)	0.2999 (3)	0.10426 (9)	0.0508 (4)	
O3	0.59059 (9)	0.0795 (2)	−0.02003 (7)	0.0394 (3)	
O4	0.58487 (8)	0.4064 (2)	−0.00286 (7)	0.0368 (3)	
O5	0.54914 (11)	0.1360 (2)	0.12874 (8)	0.0528 (4)	
O6	0.51357 (9)	0.4557 (2)	0.13481 (7)	0.0430 (4)	
C1	0.1629 (2)	0.2997 (6)	0.14566 (18)	0.0862 (11)	
H1A	0.1815	0.1822	0.1258	0.103*	
C2	0.0778 (2)	0.3155 (8)	0.1610 (2)	0.1124 (15)	
H2A	0.0396	0.2089	0.1522	0.135*	
C3	0.0512 (3)	0.4885 (8)	0.1889 (2)	0.1325 (19)	
H3A	−0.0059	0.5006	0.1990	0.159*	
C4	0.1074 (3)	0.6473 (9)	0.2026 (3)	0.1310 (18)	
H4A	0.0881	0.7654	0.2216	0.157*	
C5	0.1927 (2)	0.6303 (6)	0.18807 (18)	0.0944 (12)	
H5A	0.2312	0.7358	0.1975	0.113*	
C6	0.21971 (16)	0.4540 (4)	0.15927 (12)	0.0590 (7)	
C7	0.31009 (15)	0.4324 (4)	0.14106 (12)	0.0499 (6)	
C8	0.76481 (14)	0.4308 (4)	0.02152 (12)	0.0492 (6)	
H8A	0.7323	0.5391	0.0351	0.059*	
C9	0.85412 (16)	0.4419 (5)	0.02713 (15)	0.0660 (8)	
H9A	0.8813	0.5579	0.0444	0.079*	
C10	0.90217 (16)	0.2842 (5)	0.00760 (17)	0.0744 (9)	
H10A	0.962	0.2931	0.0110	0.089*	
C11	0.86257 (17)	0.1122 (5)	−0.01702 (18)	0.0801 (10)	
H11A	0.8957	0.0043	−0.0301	0.096*	
C12	0.77321 (15)	0.0979 (4)	−0.02254 (14)	0.0590 (7)	
H12A	0.7466	−0.0201	−0.0386	0.071*	
C13	0.72419 (13)	0.2590 (3)	−0.00417 (11)	0.0378 (5)	
C14	0.62723 (12)	0.2463 (3)	−0.01012 (9)	0.0306 (4)	
C15	0.59799 (16)	0.4938 (4)	0.26173 (12)	0.0542 (6)	
H15A	0.5636	0.6006	0.2455	0.065*	
C16	0.64396 (19)	0.5095 (5)	0.32353 (14)	0.0721 (8)	
H16A	0.6395	0.626	0.3489	0.087*	
C17	0.69539 (17)	0.3548 (6)	0.34678 (14)	0.0765 (9)	
H17A	0.7262	0.3663	0.3879	0.092*	
C18	0.70210 (18)	0.1819 (6)	0.31004 (15)	0.0743 (9)	
H18A	0.7377	0.0771	0.3261	0.089*	
C19	0.65589 (15)	0.1632 (5)	0.24906 (12)	0.0553 (6)	
H19A	0.6600	0.0453	0.2244	0.066*	
C20	0.60356 (13)	0.3198 (4)	0.22475 (10)	0.0401 (5)	
C21	0.55280 (13)	0.3005 (3)	0.15892 (10)	0.0379 (5)	
H1W1	0.4060 (18)	0.139 (5)	−0.1002 (14)	0.062 (10)*	
H2W1	0.4184 (19)	0.325 (5)	−0.1072 (15)	0.074 (10)*	
H1O	0.420 (2)	0.543 (5)	0.1603 (16)	0.090 (10)*	

### Atomic displacement parameters (Å^2^)

**Table:**

	*U*^11^	*U*^22^	*U*^33^	*U*^12^	*U*^13^	*U*^23^
Ca1	0.0308 (2)	0.0223 (2)	0.0429 (2)	−0.00138 (15)	0.00329 (15)	−0.00246 (16)
O1W	0.0509 (9)	0.0296 (9)	0.0512 (9)	0.0004 (7)	−0.0051 (7)	−0.0028 (8)
O1	0.0524 (11)	0.0671 (13)	0.0837 (13)	0.0043 (9)	0.0102 (9)	−0.0329 (11)
O2	0.0478 (9)	0.0470 (10)	0.0600 (10)	−0.0015 (7)	0.0187 (7)	−0.0103 (8)
O3	0.0376 (7)	0.0237 (8)	0.0574 (9)	−0.0041 (6)	0.0064 (6)	−0.0038 (6)
O4	0.0330 (7)	0.0254 (7)	0.0518 (8)	0.0025 (6)	0.0030 (6)	−0.0031 (6)
O5	0.0690 (10)	0.0360 (9)	0.0523 (9)	0.0049 (8)	−0.0028 (8)	−0.0066 (8)
O6	0.0437 (8)	0.0357 (9)	0.0490 (8)	0.0028 (6)	−0.0005 (6)	0.0012 (7)
C1	0.0648 (19)	0.103 (2)	0.096 (2)	−0.0124 (18)	0.0364 (17)	−0.032 (2)
C2	0.065 (2)	0.153 (4)	0.124 (3)	−0.024 (2)	0.044 (2)	−0.046 (3)
C3	0.062 (2)	0.193 (5)	0.148 (4)	0.011 (3)	0.043 (2)	−0.056 (4)
C4	0.079 (3)	0.162 (4)	0.157 (4)	0.029 (3)	0.039 (3)	−0.064 (4)
C5	0.074 (2)	0.105 (3)	0.107 (3)	0.015 (2)	0.0255 (18)	−0.039 (2)
C6	0.0508 (14)	0.0750 (19)	0.0526 (13)	0.0080 (13)	0.0136 (11)	−0.0091 (13)
C7	0.0498 (13)	0.0520 (15)	0.0491 (13)	0.0048 (11)	0.0109 (10)	−0.0039 (12)
C8	0.0369 (11)	0.0421 (14)	0.0681 (15)	−0.0041 (10)	0.0007 (10)	−0.0077 (12)
C9	0.0414 (13)	0.0679 (19)	0.0874 (19)	−0.0160 (13)	−0.0035 (12)	−0.0104 (16)
C10	0.0273 (11)	0.094 (2)	0.102 (2)	−0.0010 (14)	0.0051 (13)	−0.0049 (19)
C11	0.0390 (13)	0.083 (2)	0.119 (3)	0.0131 (15)	0.0089 (14)	−0.023 (2)
C12	0.0383 (12)	0.0518 (15)	0.0873 (19)	0.0051 (11)	0.0074 (11)	−0.0166 (14)
C13	0.0307 (10)	0.0364 (12)	0.0462 (11)	−0.0009 (8)	0.0028 (8)	0.0014 (9)
C14	0.0296 (9)	0.0256 (10)	0.0368 (10)	−0.0008 (8)	0.0045 (7)	0.0006 (8)
C15	0.0518 (13)	0.0597 (16)	0.0507 (13)	0.0025 (12)	0.0003 (10)	−0.0096 (12)
C16	0.0679 (17)	0.089 (2)	0.0579 (15)	−0.0055 (16)	−0.0038 (13)	−0.0217 (16)
C17	0.0522 (15)	0.126 (3)	0.0501 (15)	0.0002 (18)	−0.0076 (12)	0.0002 (18)
C18	0.0553 (16)	0.106 (2)	0.0605 (16)	0.0200 (17)	−0.0007 (13)	0.0175 (18)
C19	0.0498 (13)	0.0640 (17)	0.0524 (13)	0.0115 (12)	0.0053 (11)	0.0037 (13)
C20	0.0339 (10)	0.0482 (13)	0.0388 (11)	−0.0014 (9)	0.0059 (8)	0.0021 (10)
C21	0.0338 (10)	0.0386 (12)	0.0415 (11)	−0.0004 (9)	0.0053 (8)	0.0002 (9)

### Geometric parameters (Å, °)

**Table:**

O4—Ca1	2.4566 (13)	C4—C5	1.385 (5)
Ca1—O3^i^	2.3204 (14)	C4—H4A	0.9300
Ca1—O4^ii^	2.3781 (14)	C5—C6	1.382 (4)
Ca1—O1W	2.3943 (17)	C5—H5A	0.9300
Ca1—O2	2.4467 (15)	C6—C7	1.487 (3)
Ca1—O5	2.4837 (17)	C8—C13	1.382 (3)
Ca1—O6	2.5628 (15)	C8—C9	1.387 (3)
Ca1—O3	2.7414 (14)	C8—H8A	0.9300
Ca1—C21	2.892 (2)	C9—C10	1.359 (4)
Ca1—C14	2.9272 (18)	C9—H9A	0.9300
O1W—Ca1	2.3944 (17)	C10—C11	1.369 (4)
O1W—H1W1	0.75 (3)	C10—H10A	0.9300
O1W—H2W1	0.89 (3)	C11—C12	1.389 (4)
O1—C7	1.309 (3)	C11—H11A	0.9300
O1—H1O	0.92 (4)	C12—C13	1.377 (3)
O2—C7	1.217 (3)	C12—H12A	0.9300
O3—C14	1.250 (2)	C13—C14	1.506 (3)
O3—Ca1	2.7414 (14)	C14—Ca1	2.9273 (18)
O4—C14	1.261 (2)	C15—C20	1.378 (3)
O4—Ca1	2.4567 (13)	C15—C16	1.394 (3)
O5—C21	1.245 (3)	C15—H15A	0.9300
O5—Ca1	2.4838 (17)	C16—C17	1.360 (4)
O6—C21	1.272 (2)	C16—H16A	0.9300
O6—Ca1	2.5628 (15)	C17—C18	1.371 (5)
C1—C6	1.364 (4)	C17—H17A	0.9300
C1—C2	1.386 (4)	C18—C19	1.383 (4)
C1—H1A	0.9300	C18—H18A	0.9300
C2—C3	1.354 (6)	C19—C20	1.384 (3)
C2—H2A	0.9300	C19—H19A	0.9300
C3—C4	1.381 (6)	C20—C21	1.498 (3)
C3—H3A	0.9300	C21—Ca1	2.892 (2)
			
O1W—Ca1—O2	109.84 (6)	C5—C6—C7	120.6 (2)
O1W—Ca1—O3	80.16 (5)	C1—C6—C7	119.3 (3)
O1W—Ca1—O4	89.11 (5)	C1—C6—C5	120.2 (3)
O1W—Ca1—O5	151.11 (6)	O1—C7—C6	114.0 (2)
O1W—Ca1—O6	151.97 (6)	O2—C7—C6	122.8 (2)
O2—Ca1—O3	159.31 (6)	O1—C7—O2	123.3 (2)
O2—Ca1—O4	144.90 (6)	C9—C8—C13	120.0 (2)
O2—Ca1—O5	91.65 (6)	C8—C9—C10	120.4 (3)
O2—Ca1—O6	74.00 (5)	C9—C10—C11	120.1 (2)
O3—Ca1—O4	49.51 (4)	C10—C11—C12	120.4 (3)
O3—Ca1—O5	73.72 (5)	C3—C4—H4A	120.00
O3—Ca1—O6	106.02 (4)	C5—C4—H4A	120.00
O4—Ca1—O5	83.32 (5)	C4—C5—H5A	121.00
O4—Ca1—O6	75.86 (5)	C6—C5—H5A	120.00
O5—Ca1—O6	51.43 (4)	C9—C8—H8A	120.00
C11—C12—C13	119.8 (2)	C13—C8—H8A	120.00
C8—C13—C12	119.4 (2)	C8—C9—H9A	120.00
C8—C13—C14	120.15 (18)	C10—C9—H9A	120.00
C12—C13—C14	120.46 (19)	C9—C10—H10A	120.00
O4—C14—C13	118.21 (17)	C11—C10—H10A	120.00
O3—C14—O4	121.54 (17)	C10—C11—H11A	120.00
O3—C14—C13	120.22 (17)	C12—C11—H11A	120.00
C16—C15—C20	119.9 (2)	C11—C12—H12A	120.00
C15—C16—C17	120.1 (3)	C13—C12—H12A	120.00
C16—C17—C18	120.5 (3)	C16—C15—H15A	120.00
C17—C18—C19	120.0 (3)	C20—C15—H15A	120.00
C18—C19—C20	120.0 (3)	C15—C16—H16A	120.00
C15—C20—C19	119.5 (2)	C17—C16—H16A	120.00
C15—C20—C21	120.1 (2)	C16—C17—H17A	120.00
C19—C20—C21	120.4 (2)	C18—C17—H17A	120.00
O5—C21—C20	120.62 (19)	C17—C18—H18A	120.00
O6—C21—C20	118.40 (18)	C19—C18—H18A	120.00
O5—C21—O6	120.98 (19)	C18—C19—H19A	120.00
C2—C1—H1A	120.00	C20—C19—H19A	120.00
C6—C1—H1A	120.00	Ca1—O4—C14	98.75 (11)
C1—C2—H2A	120.00	O1W—Ca1—O3^i^	75.71 (6)
C3—C2—H2A	121.00	O1W—Ca1—O4^ii^	75.41 (6)
C2—C3—H3A	119.00	O2—Ca1—O3^i^	87.72 (6)
C4—C3—H3A	119.00	O2—Ca1—O4^ii^	81.92 (6)
Ca1—O2—C7	135.25 (16)	O3—Ca1—O3^i^	77.05 (5)
Ca1—O3—C14	85.74 (11)	O3—Ca1—O4^ii^	118.58 (4)
Ca1—O5—C21	95.98 (12)	O3^i^—Ca1—O4	126.34 (5)
Ca1—O6—C21	91.59 (11)	O4—Ca1—O4^ii^	74.47 (4)
C7—O1—H1O	112 (2)	O3^i^—Ca1—O5	86.45 (5)
Ca1—O1W—H1W1	119 (2)	O4^ii^—Ca1—O5	128.29 (5)
Ca1—O1W—H2W1	115 (2)	O3^i^—Ca1—O6	132.20 (5)
H1W1—O1W—H2W1	99 (3)	O4^ii^—Ca1—O6	77.79 (5)
C2—C1—C6	120.9 (4)	O3^i^—Ca1—O4^ii^	143.74 (5)
C1—C2—C3	119.0 (4)	Ca1—O3—Ca1^i^	102.95 (5)
C2—C3—C4	121.1 (4)	Ca1^i^—O3—C14	169.26 (13)
C3—C4—C5	119.9 (5)	Ca1—O4—Ca1^ii^	105.53 (5)
C4—C5—C6	119.0 (4)	Ca1^ii^—O4—C14	151.28 (12)

Symmetry codes:  (i) −*x*+1, −*y*, −*z*; (ii) −*x*+1, −*y*+1, −*z*.

### Hydrogen-bond geometry (Å, °)

**Table:**

*D*—H···*A*	*D*—H	H···*A*	*D*···*A*	*D*—H···*A*
O1W—H1W1···O5^i^	0.76 (3)	2.05 (3)	2.779 (2)	163 (3)
O1—H1O···O6	0.93 (3)	1.68 (3)	2.597 (2)	167 (3)
O1W—H2W1···O6^ii^	0.89 (3)	1.90 (3)	2.754 (2)	159 (3)

Symmetry codes: (i) −*x*+1, −*y*, −*z*; (ii) −*x*+1, −*y*+1, −*z*.

## References

[bb1] Cherkezova, V. R., Musaev, F. N. & Karaev, Z. Sh. (1987). *Russ. J. Coord. Chem.* **13**, 903–908.

[bb2] Farrugia, L. J. (1997). *J. Appl. Cryst.* **30**, 565.

[bb3] Oxford Diffraction (2007). *CrysAlis PRO* Oxford Diffraction Ltd, Abingdon, England.

[bb4] Senkovska, I. & Thewalt, U. (2005). *Acta Cryst.* C**61**, m448–m449. 10.1107/S0108270105027484 16210757

[bb5] Sheldrick, G. M. (2008). *Acta Cryst.* A**64**, 112–122. 10.1107/S0108767307043930 18156677

[bb6] Terakita, A. & Byrn, S. R. (2006). *J. Pharm. Sci.* **95**, 1162–1172. 10.1002/jps.20589 16570305

[bb7] Westrip, S. P. (2010). *J. Appl. Cryst.* **43**, 920–925.

[bb8] Yano, S., Numata, M., Kato, M., Motoo, S. & Nishimura, T. (2001). *Acta Cryst.* E**57**, m488–m490.

[bb9] Zhang, K., Yuan, J., Yuan, L. & Sun, J. (1999). *Wuhan Univ. J. Nat. Sci.* **4**, 89–94.

